# Serum Uric Acid Level Predicts Progression of IgA Nephropathy in Females but Not in Males

**DOI:** 10.1371/journal.pone.0160828

**Published:** 2016-08-25

**Authors:** Yasuyuki Nagasawa, Ryohei Yamamoto, Tatsuya Shoji, Maki Shinzawa, Yukiko Hasuike, Katsuyuki Nagatoya, Atsushi Yamauchi, Terumasa Hayashi, Takayuki Kuragano, Toshiki Moriyama, Yoshitaka Isaka, Takeshi Nakanishi

**Affiliations:** 1 Department of Internal Medicine, Division of Kidney and Dialysis, Hyogo College of Medicine 1–1 Mukogawa-Cho, Nishinomiya, Hyogo, Japan; 2 Health Care Center, Osaka University, Toyonaka, Osaka, Japan; 3 Department of Kidney Disease and Hypertension, Osaka General Medical Center, Osaka, Osaka, Japan; 4 Department of Geriatric Medicine and Nephrology, Osaka University Graduate School of Medicine, Suita, Osaka, Japan; 5 Division of Nephrology, Department of Internal Medicine, Osaka Rosai Hospital, Sakai, Osaka, Japan; University of Sao Paulo Medical School, BRAZIL

## Abstract

**Background:**

Immunoglobulin A nephropathy (IgAN) is one of most common forms of glomerulonephritis. At this point, the clinical impact of hyperuricemia on IgAN is not clear. The aim of the present study was to explore the clinical impact of hyperuricemia on the progression of IgAN.

**Study Design:**

Multicenter retrospective cohort study.

**Setting & Participants:**

935 IgAN patients who were diagnosed by kidney biopsy at Osaka University Hospital, Osaka General Hospital, and Osaka Rosai Hospital. were included in this study.

**Predictor:**

Uric acid levels at renal biopsy.

**Outcomes:**

The outcome of interest was the time from the kidney biopsy to the time when a 50% increase in the baseline serum creatinine level was observed, which was defined as "progression".

**Measurements:**

The baseline characteristics according to the kidney biopsy at the time of diagnosis were collected from the medical records, and included age, gender, body mass index, hypertension, diabetes (use of antidiabetic drugs), serum levels of creatinine, urinary protein, smoking status, RAAS blockers and steroid therapy.

**Results:**

An elevated serum uric acid level was an independent risk factor for progression in female patients (per 1.0 mg/dL, multivariate-adjusted incident rate ratio 1.33 [95% confidence interval 1.07, 1.64], P = 0.008) but not in male patients (1.02 [0.81, 1.29], P = 0.855). To control a confounding effect of renal function on an association between serum uric acid level and progression in female patients, age- and serum creatinine-matched and propensity score-matched analyses were performed, and these results also supported the effect by uric acid on kidney disease progression independent of basal kidney function.

**Limitations:**

A cohort analyzed retorospectively.

**Conclusions:**

This study revealed that an elevated uric acid level was an independent risk factor for ESKD in female IgAN patients. Therefore, uric acid might be a treatable target in female IgAN patients.

## Introduction

Immunoglobulin A nephropathy (IgAN) is one of most common forms of glomerulonephritis [1], and the 30-year renal survival rate has been reported to be 50.3% [2], which indicates that its prognosis is poor. Several types of factors are related to the pathogenesis of IgAN, such as genetic factors [3,4], immune factors [5–7], and infectious factors [8,9]. In addition to the classical renal progression factors, including hypertension, proteinuria, and decreased renal function, other factors that are related to the renal progression of IgAN have been reported, such as smoking [10,11], hypertriglyceridemia [12], hyperuricemia [12], and atherosclerosis-related genetic factors [4,10,13,14].

Serum uric acid level has been considered to be an atherosclerotic factor [15–18]. Moreover, many reports have suggested that females are at a higher risk for uric acid-induced atherosclerotic diseases than males, whereas no evidence indicates that males experience more effects from uric acid than females. Indeed, associations that have been found between the serum uric acid level and the incidence of coronary heart disease [19], hypertension [17], and kidney dysfunction [20,21] were stronger in women than in men. Uric acid is secreted in the kidney, and the deterioration of kidney function should lead to high uric acid levels. Therefore, if the association between the glomerular filtration rate (GFR) and uric acid was not included in a given analysis, the effect of uric acid on renal function might have been overestimated. To support the idea that uric acid may be a predictor of renal function in patients with IgAN, a Finnish IgAN cohort study reported that the IgAN group with progression of renal disease had a higher baseline serum uric acid level than those in the IgAN group with stable renal function. However, the baseline renal function was not included in many statistical analyses [12]. Moreover, the authors of this report did not determine the gender differences with respect to the uric acid level, even though females are expected to be at a higher risk from the effects of uric acid than males. At this point, the clinical impact of hyperuricemia on IgAN is not clear.

The aim of the present study was to explore the clinical impact of hyperuricemia on the progression of IgAN. This multicenter observational cohort study was organized by a research group for the Study of Outcomes and Practice patterns of primary Immunoglobulin A Nephropathy (STOP-IgAN), which is based out of three major nephrology centers in Osaka, Japan.

## Methods

### Patients

The candidate patients of the present study were included in our previous retrospective cohort study: the Study of Outcomes and Practice patterns of IgA Nephropathy (STOP-IgAN). The study protocol was described in detail elsewhere [13]. Briefly, between 1992 and 2005, 1001 patients aged at least 15 years were diagnosed with IgAN by kidney biopsy at Osaka University Hospital, Osaka General Hospital, and Osaka Rosai Hospital. The informed consent for the kidney biopsy, on behalf of patients between 15 to 20, was always done accompanied with their parent, and written informed consent for kidney biopsy was obtained from parents. This procedure obeyed the Japanese Low. After the exclusion of 29 (2.9%) patients who were treated with uric acid-lowering agents at the time of kidney biopsy and 37 (3.7%) patients with missing data, the present study finally included 935 (93.4%) patients. The patients were followed-up until June 2009. The study protocol was approved by the ethics committees (Osaka University Hospital Ethical Committee, Osaka Rousai Hosipital Ethical Committee, Osaka general medical center ethical committee) at the three hospitals (Osaka University Hospital, Osaka Rousai Hospital, Osaka general medical center). The procedure for informed consent was that our study protocol was open to public in our homepage (http://www.med.osaka-u.ac.jp/pub/kid/kid/studyPlan/stopigan.htm), and if the participants would not want to be included in this study, they can contact with us. There was no patient who wanted not to be included in this study. We used the clinical renal biopsy database which each hospital had for clinical use to build up the IgA nephropathy cohort in each hospital. The all data were de-identified with linking capacity within each facility before the data from three hospitals were combined and authors made statistical analysis. We cut the patient name, the day of birth (except month, year of birth), patient ID in each hospital, labeled the patients in each hospital, and made the correspondence table and the table was kept in the locked area. Data are available from the Osaka University Institutional Data Access / Ethics Committee for researchers who meet the criteria for access to confidential data (rinri@hp-crc.med.osaka-u.ac.jp, Osaka University Hospital, Future medical Center, Division of clinical Trial, Yamadaoka2-2, Suita, Osaka, Japan)

### Measurements

The baseline characteristics according to the kidney biopsy at the time of diagnosis were collected from the medical records, and included age, gender, body mass index, hypertension (systolic blood pressure ≥ 140 mmHg, diastolic blood pressure ≥ 90 mmHg, and/or use of antihypertensive drugs), diabetes (use of antidiabetic drugs), serum levels of creatinine and uric acid, urinary protein, smoking status (current, past, or non-smokers), and the number of cigarettes smoked daily at the time of the kidney biopsy (0 for past smokers and non-smokers). At two hospitals, the level of serum creatinine was measured by the enzymatic method during the entire observational period, whereas the measurement of creatinine levels was changed from the Jaffe method to the enzymatic method in November 1995 at one hospital. Using sera from 648 patients who were randomly selected from the hospital, the correlation coefficient r between creatinine values that was measured by the Jaffe method and the enzymatic method was 0.998; the least squares method determined the following predictive equation: creatinine (enzymatic method) = 0.94 X creatinine (Jaffe method)—0.25. Thus, the creatinine values for the 205 patients who were diagnosed with IgAN by kidney biopsy before November 1995 at the three hospitals were calibrated using this equation. The antihypertensive drugs that were used by the patients were as follows: RAAS blockers (angiotensin-converting enzyme inhibitors, angiotensin II receptor blockers, aldosterone receptor blockers), calcium channel blockers, alpha-blockers, beta-blockers, and thiazides. Information on the smoking status and the number of cigarettes smoked daily was obtained via a questionnaire when the patients were admitted to the hospital for the kidney biopsy.

Information on the use of RAAS blockers and corticosteroids initiated within one year of the kidney biopsy was also collected to determine any therapeutic interventions.

### Outcome

The outcome of interest was the time from the kidney biopsy to the time when a 50% increase in the baseline serum creatinine level was observed, which was defined as "progression". Serum creatinine was measured as required according to the clinical needs of each patient. The patients were followed-up until June 2009 and were censored on the last day of measurement of serum creatinine before June 2009.

### Statistics

**Fig 1:**
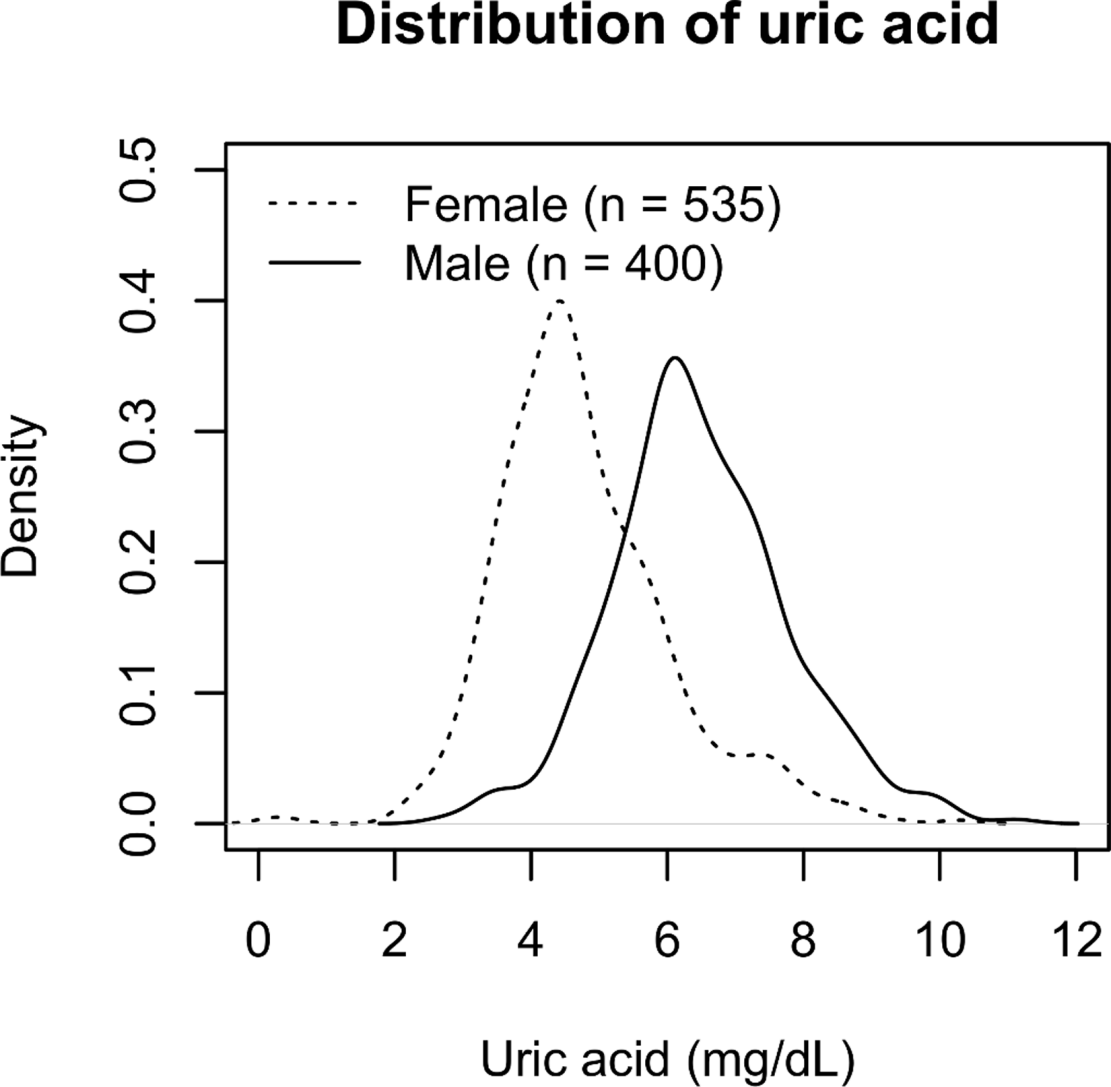
Kernel density estimates of serum uric acid levels after kidney biopsy of 535 female and 400 male patients.

The distributions of the serum uric acid levels in the male and female patients were described with the kernel density estimation and were compared using unpaired t-test. Because the male and female distributions of uric acid were substantially different (Fig 1), all subsequent analyses were conducted separately in males and females. After the categorization of the baseline uric acid level into male and female quartiles, the differences in their clinical characteristics were compared using ANOVA, the Kruskal-Wallis test or the chi-square test, as appropriate. The cumulative probability of progression was calculated with the Kaplan-Meier method. An association between the serum uric acid level and progression was assessed by log-rank test and the Poisson regression model, which were adjusted for clinically relevant factors. The appropriateness of the Poisson regression model was tested with a goodness-of-fit test using deviance statistics.

To control the confounding effect of renal function on an association between serum uric acid level and progression, each patient in the first (Q1) and second (Q2) quartiles of serum uric acid level at kidney biopsy were randomly matched with a patient in its third (Q3) and fourth (Q4) quartiles at a ratio of 1:1 without replacement, using two distinct matching methods. In the first method, patients were matched by baseline age (±5 years) and serum creatinine (±0.1 mg/dL). The second method used a propensity score, an estimated probability of being a Q3 –Q4 patient, given the observed confounding variables [22]. The propensity score for each patient was calculated in a separate multivariate logistic regression model including the baseline confounding factors (age, body mass index, hypertension, serum creatinine level, urinary protein excretion, the number of cigarettes smoked daily, and use of antidiabetic drugs) and use of RAAS blockers and corticosteroids initiated within one year of the kidney biopsy as independent variables. Calibration was assessed using Hosmer-Lemeshow goodness-of-fit test. Each Q1 –Q2 patient were matched to Q3 –Q4 patient with the closest propensity score, using a greedy matching algorithm with a caliper width of 0.2 standard deviation of the logit the propensity score [23]. In the age- and serum creatinine-matched pairs and the propensity score-matched pairs, baseline characteristics at kidney biopsy and therapeutic interventions within 1 year of kidney biopsy were compared, using paired t-test, Wilcoxon singed rank test, or McNemar test, as appropriate. Incident ratio ratio (IRR) of Q3 –Q4 of serum uric acid level (vs. Q1 –Q2) was calculated using Poisson regression model.

Statistical significance was set at P < 0.05. Statistical analyses were performed using R version 3.2.4 "Very Secure Dishes" (The R Foundation for Statistical Computing, www.r-project.org).

## Results

The mean baseline serum uric acid levels of the 535 female and 400 male patients at the time of diagnosis by kidney biopsy were 4.8±1.3 (mean±SD) and 6.5±1.3 mg/dL, respectively (P < 0.001) (Fig 1). Their clinical characteristics that were stratified into quartiles of the baseline serum uric acid level are described in Tables 1 and 2, respectively. In female patients, a significant difference was observed in the following parameters among quartiles of the baseline serum uric acid level: age, body mass index, hypertension, serum creatinine, urinary protein, the number of cigarettes smoked daily by current smokers at the time of diagnosis by kidney biopsy, and therapeutic interventions (RAAS blockade and the use of corticosteroids) within one year of kidney biopsy (Table 1). In male patients, similar differences were observed, except in the baseline number of cigarettes smoked daily by current smokers and therapeutic interventions (RAAS blockade within 1 year of kidney biopsy) (Table 2).

**Table 1:** Clinical characteristics of 535 female patients with IgAN stratified in quartiles of uric acid levels.

	Q1	Q2	Q3	Q4	P
Uric acid (mg/dL)	3.6 (0.2–3.9)	4.3 (4.0–4.6)	5.0 (4.7–5.4)	6.2 (5.5–10.3)	
Number	140	146	118	131	
Baseline characteristics at renal biopsy					
Age (years)	27 (22, 37)	28 (23, 44)	29 (22, 45)	44 (30, 53)	<0.001
Body mass index (kg/m^2^)	20.6±2.7	21.0±3.0	21.9±3.9	24.0±3.9	<0.001
Hypertension (%)^§^	10.0	18.5	21.2	49.6	<0.001
Serum creatinine (mg/dL)	0.60 (0.51, 0.69)	0.60 (0.59, 0.70)	0.68 (0.60, 0.80)	0.85 (0.70, 1.07)	<0.001
Urinary protein (g/day)	0.24 (0.13, 0.53)	0.30 (0.16, 0.62)	0.44 (0.18, 0.86)	0.64 (0.32, 1.70)	<0.001
Current smoker (%)	12.1	14.4	12.7	16.0	0.448
Cigarettes (per day)^#^	6 (5, 10)	15 (10, 20)	15 (10, 20)	20 (10, 20)	0.297
Diabetes (%)	0.8	0.7	0.8	0.8	0.927
Therapeutic interventions within 1 year of kidney biopsy					
RAAS blockade (%)	25.0	21.9	40.7	60.3	<0.001
Use of corticosteroids (%)	18.6	28.8	41.5	26.0	0.047

Median (min—max), median (25%, 75%), mean±SD.

RAAS, renin-angiotensin aldosterone system

^§^Systolic blood pressure ≥140 mmHg, diastolic blood pressure ≥90 mmHg, and/or use of antihypertensive drugs at the time of kidney biopsy

^#^Number of cigarettes smoked in current smokers at the time of kidney biopsy was displayed. P value was calculated after including past and non-smokers with 0 cigarette per day.

**Table 2:** Clinical characteristics of 400 male patients with IgAN stratified in quartiles of uric acid levels.

	Q1	Q2	Q3	Q4	P
Uric acid (mg/dL)	5.2 (2.7–5.7)	6.1 (5.8–6.4)	6.8 (6.5–7.2)	8.0 (7.3–11.1)	
Number	104	108	88	100	
Baseline characteristics at renal biopsy					
Age (years)	28 (20, 47)	28 (21, 46)	30 (22, 45)	37 (26, 52)	0.002
Body mass index (kg/m^2^)	21.6±2.9	22.8±3.2	23.0±3.2	24.3±3.6	<0.001
Hypertension (%)^§^	28.8	32.4	36.4	47.0	0.006
Serum creatinine (mg/dL)	0.80 (0.73, 0.90)	0.87 (0.73, 1.00)	0.90 (0.78, 1.01)	1.07 (0.90, 1.20)	<0.001
Urinary protein (g/day)	0.31 (0.15, 0.68)	0.37 (0.19, 0.73)	0.41 (0.22, 0.96)	0.62 (0.36, 1.35)	<0.001
Current smoker (%)	31.7	29.6	29.5	36.0	0.530
Cigarettes (per day)^#^	20 (10, 25)	20 (15, 26)	20 (10, 20)	20 (20, 33)	0.350
Diabetes (%)	1.9	0.9	1.1	4.0	0.292
Therapeutic interventions within 1 year of kidney biopsy					
RAS blockade (%)	44.2	51.9	44.3	59.0	0.092
Use of corticosteroids (%)	18.3	32.4	21.6	34.0	0.060

Median (min—max), median (25%, 75%), mean±SD.

RAS, renin-angiotensin system

^§^Systolic blood pressure ≥140 mmHg, diastolic blood pressure ≥90 mmHg, and/or use of antihypertensive drugs at kidney biopsy

^#^Number of cigarettes smoked in current smokers at the time of kidney biopsy was displayed. P value was calculated after including past and non-smokers with 0 cigarette per day.

**Fig 2:**
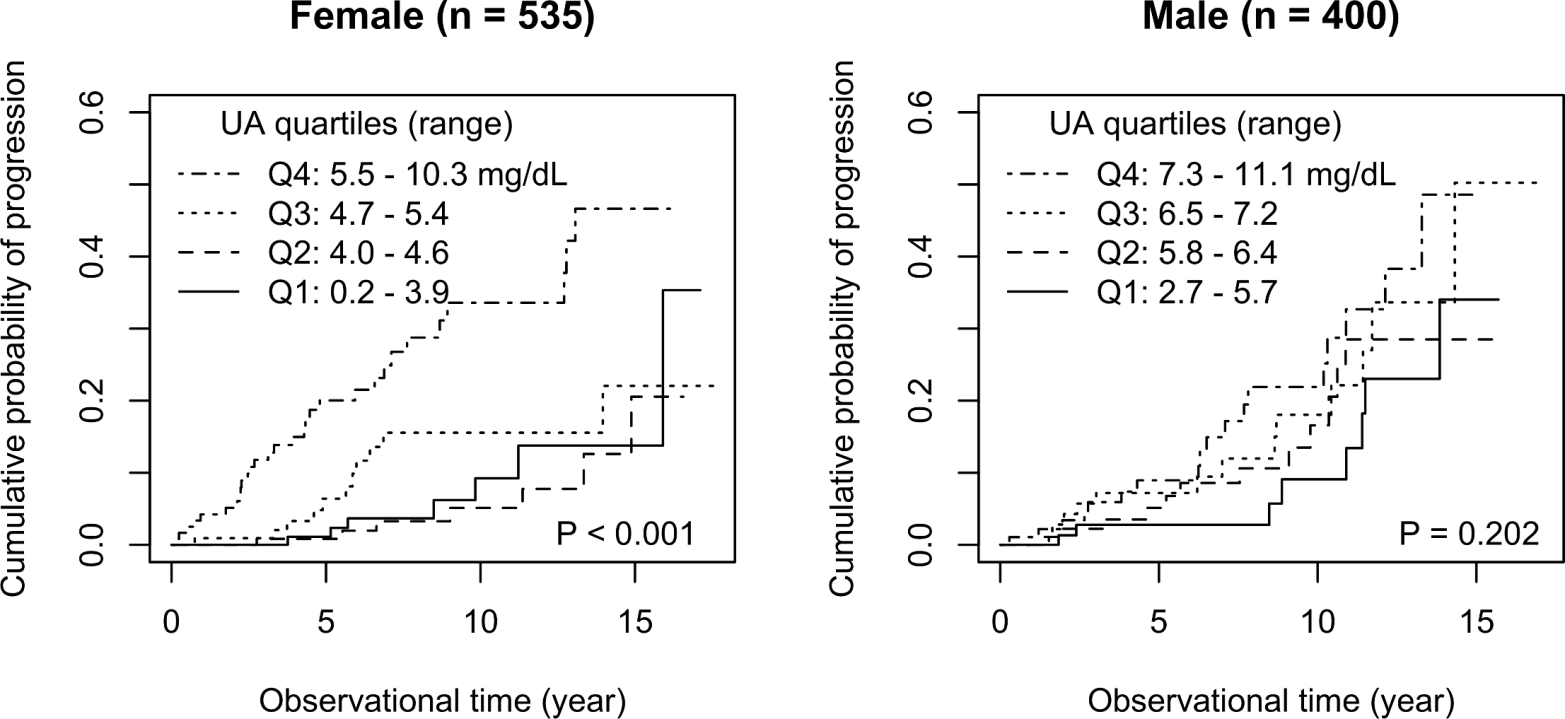
Cumulative probabilities of a 150% increase in the serum creatinine level (progression) in 535 female and 400 male patients who were stratified into quartiles based on uric acid level.

In female patients, during the median 5.7 years (interquartile range 2.6, 9.3) of the observational period, 7 (5.0%), 7 (4.8%), 11 (9.3%), and 30 (22.9%) patients in Q1, Q2, Q3, and Q4 of serum uric acid levels developed progression, respectively (P < 0.001). Female patients in the higher quartiles of serum uric acid levels were at a higher risk of progression (the cumulative probabilities of progression at 5 and 10 years were 0.01 [95% confidence interval 0.00, 0.03] and 0.09 [0.00, 0.17] in Q1 patients, 0.01 [0.00, 0.02] and 0.05 [0.00, 0.10] in Q2 patients, 0.06 [0.01, 0.12] and 0.16 [0.06, 0.24] in Q3 patients, and 0.20 [0.12, 0.28] and 0.34 [0.21, 0.44] in Q4 patients, respectively (P < 0.001)) (Fig 2). On the contrary, in male Q1, Q2, Q3, and Q4 patients, progression was observed in 8 (7.7%), 12 (11.1%), 13 (14.8%), and 18 (18.0%) patients, respectively, during the 5.2 years (interquartile range 2.1, 9.8) of the observational period (P = 0.141). No significant difference was observed in the cumulative probabilities of progression among Q1, Q2, Q3, and Q4 patients (cumulative probabilities of progression at 5 and 10 years was 0.03 [95% confidence interval 0.00, 0.06] and 0.09 [0.00, 0.18] in Q1 patients, 0.05 [0.00, 0.10] and 0.17 [0.05, 0.27] in Q2 patients, 0.07 [0.01, 0.13] and 0.18 [0.06, 0.29] in Q3 patients, and 0.09 [0.02, 0.15] and 0.22 [0.10, 0.32] in Q4 patients, respectively (P = 0.202)) (Fig 2).

In a univariate Poisson regression model, the serum uric acid level was significantly associated with progression in female patients (per 1.0 mg/dl, IRR 1.82 [95% confidence interval 1.54, 2.13], P < 0.001) (Table 3). Even after adjustment for clinically relevant factors, an association between the serum uric acid level and progression was still significant, although its association was attenuated (1.33 [1.07, 1.64], P = 0.008 in multivariate model 1; 1.34 [1.05, 1.68], P = 0.015 in model 2 in Table 3). An elevated serum uric acid level was identified to be a significant predictor of progression in female patients, together with older age, and elevated levels of serum creatinine and proteinuria. In regard to male patients, the serum uric acid level was associated with progression at a marginally significant level according to a univariate model (1.20 [0.97, 1.47], P = 0.089). However, a multivariate adjustment for clinically relevant factors blunted this association (1.02 [0.81, 1.29], P = 0.855 in model 1; 1.04 [0.80, 1.34], P = 0.787 in model 2 in Table 3). Elevated serum levels of serum creatinine and proteins in the urine, the number of cigarettes smoked daily, and the presence of diabetes were identified to be significant predictors of progression in male patients.

**Table 3:** Predictors of progression in 535 female and 400 male patients with IgAN.

	Univariate model		Multivariate model 1		Multivariate model 2	
	IRR (95%CI)	P	IRR (95%CI)	P	IRR (95%CI)	P
Predictors in female patients (n = 533)						
Age (per 10 years)	1.53 (1.27, 1.85)	<0.001	1.27 (1.02, 1.57)	0.032	1.29 (1.02, 1.62)	0.035
Body mass index (per 1.0 kg/m^2^)	1.05 (0.98, 1.12)	0.121			0.97 (0.88, 1.05)	0.445
Hypertension	3.24 (1.91, 5.52)	<0.001	1.32 (0.71, 2.48)	0.380	1.30 (0.68, 2.46)	0.423
Serum creatinine (per 1.0 mg/dL)	4.07 (3.15, 4.98)	<0.001	2.60 (1.80, 3.48)	<0.001	2.72 (1.85, 3.73)	<0.001
Urinary protein (per 1.0 g/day)	1.44 (1.27, 1.60)	<0.001	1.20 (1.03, 1.37)	0.008	1.26 (1.07, 1.46)	0.003
Uric acid (per 1.0 mg/dL)	1.82 (1.54, 2.13)	<0.001	1.33 (1.07, 1.64)	0.008	1.34 (1.05, 1.68)	0.015
Cigarettes (per 20/day)^#^	1.85 (0.76, 3.83)	0.130			1.39 (0.63, 2.63)	0.361
Diabetes	2.13 (0.12, 9.67)	0.454			1.25 (0.07, 6.21)	0.832
RAS blockade	1.96 (1.15, 3.34)	0.013			0.86 (0.46, 1.60)	0.632
Use of corticosteroids	0.92 (0.48, 1.65)	0.792			0.61 (0.29, 1.21)	0.178
Predictors in male patients (n = 400)						
Age (per 10 years)	1.21 (1.00, 1.47)	0.048	1.09 (0.88, 1.34)	0.427	0.91 (0.73. 1.15)	0.424
Body mass index (per 1.0 kg/m^2^)	1.01 (0.93, 1.09)	0.812			0.96 (0.87, 1.07)	0.482
Hypertension	1.52 (0.88, 2.65)	0.133	1.15 (0.64, 2.07)	0.637	1.17 (0.62, 2.21)	0.623
Serum creatinine (per 1.0 mg/dL)	3.24 (1.75, 5.07)	<0.001	2.36 (1.14, 4.17)	0.008	2.89 (1.34, 5.65)	0.003
Urinary protein (per 1.0 g/day)	1.44 (1.25, 1.63)	<0.001	1.39 (1.20, 1.59)	<0.001	1.42 (1.20, 1.66)	<0.001
Uric acid (per 1.0 mg/dL)	1.20 (0.97. 1.47)	0.089	1.02 (0.81, 1.29)	0.855	1.04 (0.80, 1.34)	0.787
Cigarettes (per 20/day)^#^	1.90 (1.39, 2.53)	<0.001			1.88 (1.31, 2.63)	<0.001
Diabetes	7.28 (2.20, 17.88)	<0.001			8.88 (2.48, 24.91)	<0.001
RAS blockade	1.22 (0.70, 2.17)	0.496			0.90 (0.48, 1.69)	0.737
Use of corticosteroids	0.79 (0.41, 1.45)	0.466			0.56 (0.26, 1.11)	0.108

CI, confidence interval; IRR, incident rate ratio.

To clarify an association between the serum uric acid level and progression, IRR of each quartile of serum uric acid levels was calculated. In females, compared with Q2, Q4 was significantly associated with progression in a univariate model (Q1, 1.26 [0.43, 3.69], P = 0.662; Q2, 1.00 [reference]; Q3, 2.25 [0.89, 6.11], P = 0.094; Q4, 6.16 [2.87, 15.27], P < 0.001) (univariate model in Table 4). Even after an adjustment for age, hypertension, serum creatinine, and urinary protein, Q4 was significantly associated with progression, but these covariates greatly attenuated the magnitude of the association between the level of serum uric acid and progression (Q1, 1.40 [0.48, 4.09], P = 0.533; Q2, 1.00 [reference]; Q3, 1.82 [0.71, 4.98], P = 0.217; Q4, 2.56 [1.10, 6.70], P = 0.039) (multivariate model 1 in Table 4). Further adjustment for body mass index, the number of cigarettes, the presence of diabetes at the time of kidney biopsy, and therapeutic interventions showed a similar result (multivariate model 2 in Table 4). In male patients, no significant association was observed between the quartiles of serum uric acid levels and progression (Table 4).

**Table 4:** Associations between uric acid and progression.

	Univariate model		Multivariate model 1^†^		Multivariate model 2^‡^	
	IRR (95%CI)	P	IRR (95%CI)	P	IRR (95%CI)	P
Quartiles of uric acid levels in female patients (n = 533)						
Q1: 3.6 (0.2–3.9) mg/dL	1.26 (0.43, 3.69)	0.662	1.40 (0.48, 4.09)	0.533	1.30 (0.44, 3.86)	0.624
Q2: 4.3 (4.0–4.6)	1.00 (reference)		1.00 (reference)		1.00 (reference)	
Q3: 5.0 (4.7–5.2)	2.25 (0.89, 6.11)	0.094	1.82 (0.71, 4.98)	0.217	1.99 (0.77, 5.52)	0.163
Q4: 6.2 (5.8–7.2)	6.16 (2.87, 15.27)	<0.001	2.56 (1.10, 6.70)	0.039	2.58 (1.05, 7.05)	0.049
Quartiles of uric acid levels in male patients (n = 400)						
Q1: 5.2 (2.7–5.7) mg/dL	0.78 (0.30, 1.88)	0.580	0.92 (0.36, 2.25)	0.854	0.85 (0.32, 2.14)	0.727
Q2: 6.1 (5.8–6.2)	1.00 (reference)		1.00 (reference)		1.00 (reference)	
Q3: 6.8 (6.5–7.2)	1.33 (0.60, 2.96)	0.475	1.26 (0.57, 2.79)	0.570	1.45 (0.65, 3.30)	0.363
Q4: 8.0 (7.3–11.1)	1.64 (0.80, 3.49)	0.185	0.99 (0.46, 2.20)	0.984	1.10 (0.48, 2.53)	0.826

Median (min—max)

CI, confidence interval; IRR, incident rate ratio

^†^Adjusted for age, hypertension, serum creatinine, and urinary protein at the time of kidney biopsy.

^‡^Adjusted for the covariates in model 1 plus body mass index, the number of cigarettes, diabetes at kidney biopsy, renin-angiotensin system blockade and the use of corticosteroids within one year of kidney biopsy.

To control a confounding effect of renal function on an association between serum uric acid level and progression in female patients, 173 patients in Q1 and Q2 were randomly matched to 173 patients in Q3 and Q4 by age (±5 years) and serum creatinine level (±0.1 mg/dL) (Table 5). Significant differences were observed in baseline body mass index, serum creatinine level, and urinary protein, and use of corticosteroid within 1 year of kidney biopsy, although serum creatinine level was, clinically, not at significant level (Q1 –Q2, median 0.60 mg/dL [interquartile range 0.58, 0.72] vs. Q3 –Q4, 0.68 [0.60, 0.78], P <0.001). Compared with Q1 –Q2, Q3 –Q4 was significantly associated with progression in univariate model (IRR 2.87 [1.18, 8.00], P<0.001), multivariate model 1 (2.69 [1.06, 7.70], P = 0.046), and multivariate model 2 (2.97 [1.07–9.31], P = 0.046) similarly (Table 6).

**Table 5:** Clinical characteristics of age- and serum creatinine-matched female patients (n = 346).

	Q1—Q2	Q3—Q4	P
Uric acid (mg/dL)	4.0 (0.2–4.6)	5.3 (4.7–8.6)	
Number	173	173	
Baseline characteristics at renal biopsy			
Age (years)	29 (23, 43)	29 (22, 44)	0.713
Body mass index (kg/m^2^)	20.7±3.0	22.7±4.3	<0.001
Hypertension (%)^§^	18.5	21.4	0.551
Serum creatinine (mg/dL)	0.60 (0.58, 0.72)	0.68 (0.60, 0.78)	<0.001
Urinary protein (g/day)	0.30 (0.13, 0.60)	0.46 (0.20, 0.95)	<0.001
Current smoker (%)	12.7	13.9	0.871
Cigarettes (per day)^#^	10 (5, 20)	15 (10, 20)	0.406
Diabetes (%)	1.0	0.0	
Therapeutic interventions within 1 year of kidney biopsy			
RAAS blockade (%)	23.1	45.7	<0.001
Use of corticosteroids (%)	25.4	34.1	0.919

Median (min—max), median (25%, 75%), mean±SD.

RAAS, renin-angiotensin aldosterone system

^§^Systolic blood pressure ≥140 mmHg, diastolic blood pressure ≥90 mmHg, and/or use of antihypertensive drugs at the time of kidney biopsy

^#^Number of cigarettes smoked in current smokers at the time of kidney biopsy was displayed. P value was calculated after including past and non-smokers with 0 cigarette per day.

**Table 6:** Associations between uric acid and progression in matched female patients.

		Quartiles of baseline uric acid		
		Q1—Q2	Q3—Q4	
Age- and serum creatinine-matched patients (n = 346)				
Baseline uric acid (mg/dL)		4.0 (0.2–4.6)	5.3 (4.7–8.6)	
Univariate model	IRR (95% CI)	1.00 (reference)	2.87 (1.18, 8.00)	<0.001
Multivariate model 1^†^		1.00 (reference)	2.69 (1.06, 7.70)	0.046
Multivariate model 2^‡^		1.00 (reference)	2.97 (1.07, 9.31)	0.046
Propensity score-matched patients (n = 284)				
Baseline uric acid (mg/dL)		4.1 (2.2–4.6)	5.3 (4.7–9.3)	
Univariate model	IRR (95% CI)	1.00 (reference)	3.09 (1.18, 9.57)	0.030

Median (min—max)

CI, confidence interval; IRR, incident rate ratio

^†^Adjusted for age, hypertension, serum creatinine, and urinary protein at the time of kidney biopsy.

^‡^Adjusted for the covariates in model 1 plus body mass index, the number of cigarettes, diabetes at kidney biopsy, renin-angiotensin system blockade and the use of corticosteroids within one year of kidney biopsy.

Between propensity score-matched pairs (n = 284), no significant difference was observed in baseline characteristics and therapeutic interventions within 1 year of kidney biopsy (Table 7). Even after propensity score-matching, Q3 –Q4 patients were at significantly higher risk of progression, compared with Q1 –Q2 patients (IRR 3.09 [1.18, 9.57], P = 0.030) (Table 6).

**Table 7:** Clinical characteristics of propensity score-matched patients (n = 284).

	Q1–2	Q3–4	P
Uric acid (mg/dL)	4.1 (2.2–4.6)	5.3 (4.7–9.3)	
Number	142	142	
Baseline characteristics at renal biopsy			
Age (years)	31 (24, 44)	31 (23, 46)	0.515
Body mass index (kg/m^2^)	21.6±3.2	21.8±3.3	0.514
Hypertension (%)^§^	23.9	21.1	0.671
Serum creatinine (mg/dL)	0.60 (0.59, 0.75)	0.67 (0.60, 0.78)	0.231
Urinary protein (g/day)	0.37 (0.16, 0.70)	0.39 (0.19, 0.70)	0.580
Current smoker (%)	17.6	12.0	0.256
Cigarettes (per day)^#^	10 (5, 20)	15 (10, 20)	0.449
Diabetes (%)	0.7	0.0	
Therapeutic interventions within 1 year of kidney biopsy			
RAAS blockade (%)	35.9	36.6	1.000
Use of corticosteroids (%)	27.5	29.6	0.787

Median (min—max), median (25%, 75%), mean±SD.

RAAS, renin-angiotensin aldosterone system

^§^Systolic blood pressure ≥140 mmHg, diastolic blood pressure ≥90 mmHg, and/or use of antihypertensive drugs at the time of kidney biopsy

^#^Number of cigarettes that were smoked daily by current smokers at the time of kidney biopsy was displayed. P value was calculated after including past and non-smokers with 0 cigarette per day.

## Discussion

This retrospective observational study of 935 patients with IgAN revealed that an elevated serum uric acid level was an independent risk factor for progression in female patients (per 1.0 mg/dL, multivariate-adjusted IRR 1.33 [95% confidence interval 1.07, 1.64], P = 0.008) but not in male patients (1.02 [0.81, 1.29], P = 0.855). To control a confounding effect of renal function on an association between serum uric acid level and progression in female patients, age- and serum creatinine-matched and propensity score-matched analyses were performed, and these results also supported the effect by uric acid on kidney disease progression independent of basal kidney function. Two of the advantages of the present study were a very large cohort and a long observational period. Those advantages enabled us to assess an association between the serum uric acid level and the progression of IgAN in male and female patients separately, after adjustment for clinically relevant factors including the serum creatinine level, which is an index of renal function.

A limited number of cohort studies have reported an association between the serum uric acid level and progression of IgAN. A Finnish cohort study first showed that hyperuricemia (defined as > 7.56 mg/dl in males and > 5.55 mg/dl in females) was a risk factor for the progression of IgAN, but this result had not been adjusted for kidney function [12]. Ohno et al reported that uric acid impacted renal prognosis in IgA nephropathy but did not provide an analysis of the gender differences [24]. Shi-Y et al also confirmed that uric acid was a risk factor for progression in 353 patients with IgA nephropathy in a retrospective cohort, but again, an analysis of the gender differences was not included [25]. Recently, uric acid was determined to be a risk factor for renal dysfunction only in patients with IgAN with stage G3a CKD [26]. Our study revealed the effect of uric acid upon disease progression in female patients with IgAN.

Our results indicated a gender difference with respect to the effects of uric acid on the progression of uric acid-induced kidney disease. The mechanism of this gender difference in the association between the serum uric acid level and the progression of IgAN remains unclear. Estrogen suppressed the protein levels of the urate reabsorptive transporter (urate transporter 1; URAT1) in the kidney, which resulted in an increase in uric acid excretion and a decrease in the uric acid level in the serum [27]. Indeed, postmenopausal women have higher serum uric acid levels, and the use of postmenopausal hormones (e.g., estrogen analogs) is associated with lower levels of uric acid [28]. After natural menopause, women experience an increase in uric acid levels and an increase in the risk of atherosclerotic diseases [29,30]. Female IgAN patients with high uric acid levels might have a similar atherosclerotic condition as menopausal women with high levels of uric acid and low levels of estrogen. Moreover, renal arteriolar hyalinosis could be significantly highly observed in female subjects with a uric acid level of more than 5 mg/dl, whereas the hyalinosis lesions were observed in male subjects with a uric acid level of more than 7 mg/dl [31]. These findings suggest that females might be more vulnerable to uric acid-induced organ damage, which might coincide with the phenomenon that uric acid levels in females were within a lower range than those in males. In terms of anti-arteriosclerosis therapy, use of satin for IgA nephropathy female patients with hyper uric acid might be useful. Many report including meta-analysis supported the renal protective effect of statin [32,33]. While information about the statin use was not available, average age of IgA nephropathy cohort in this study was 31years old, and average total cholesterol was 194mg/dl (5.03mmol/L), therefore, major part of this cohort might be not treated with statin. And also there were very few atherosclerotic events to detect the gender differences of CVD events due to relative young age of this cohort, although some reports support gender difference of CVD events associated hyper uric acids [17,19–21].

Several interventional studies have demonstrated that the treatment of hyperuricaemia ameliorated the progression of CKD [18]. A Chinese randomized controlled trial that included 54 hyperuricemic patients with CKD, demonstrated that allopurinol decreased systolic blood pressure and slowed the progression of CKD [18]. Through a non-randomized interventional study, Kanbay et al also reported this favorable effect of uric acid-lowering therapy on blood pressure and GFR in patients with stage G2 CKD [34]. Additionally, the treatment of 40 IgA nephropathy patients with allopurinol decreased their blood pressure, although the small number of patients and the short observational period hindered a statistically meaningful assessment of any renoprotective effect of allopurinol [25]. This reduction in the progression of CKD by uric acid-lowering therapy was considered to be induced by an improvement in endothelial function [15,18]. Endothelial cells are one of the important cell types in the glomeruli, and they are considered to be related to hypertension and renal progression [35]. Our results suggested that hyperuricemia might be a treatable target in patients with IgAN, especially in female patients.

Our study has several limitations. First, this study did not include information in regard to uric acid-lowering therapy during the observational periods, although patients with IgAN who received this therapy at the time of renal biopsy were excluded from this study. The majority of patients in this study had uric acid levels that were lower than those in the treatable range; therefore, this effect might be small. Second, although all patients were diagnosed with IgAN by kidney biopsy, histological findings were not included as covariates of interest in the present study, partly because each facility used different histological grading systems; each facility also changed their grading system during the 14 years of the long entry period between 1992 and 2005. Recently, plasma uric acid reported to have association with interstitial fibrosis and tubular atrophy in IgA nephropathy patients [36], while this report did not demonstrate histological gender difference. The possibility that effect upon kidney dysfunction caused by uric acid may be mediated by kidney histological change should be confirmed by further study. Third, this study did not include the alcohol drinking status of the patients. The PREVEND study reported that alcohol consumption is inversely associated with the risk of development of CKD [37]. In Japan, alcohol consumption in males was higher than that in females, and therefore, the renoprotective effect of alcohol might lead to improvement in renal prognosis and may elevate the levels of uric acid in male patients with IgA nephropathy.

In conclusion, this study revealed that an elevated uric acid level was an independent risk factor for ESKD in female IgAN patients. Therefore, uric acid might be a treatable target in female IgAN patients.
